# Global research trends and prospects related to tumor microenvironment within Triple Negative Breast Cancer: a bibliometric analysis

**DOI:** 10.3389/fimmu.2023.1261290

**Published:** 2023-12-04

**Authors:** Peiting Li, Jun Li, Xiaofei Tong, Zhenyang Xiao, Wuliang Diao, Chi Zhong, Jianda Zhou, Wei Wu

**Affiliations:** ^1^ Department of Plastic Surgery, The Third Xiangya Hospital, Central South University, Changsha, China; ^2^ Department of Breast Thyroid Surgery, The Third Xiangya Hospital, Central South University, Changsha, China

**Keywords:** tumor microenvironment, Triple Negative Breast Cancer, CiteSpace, VOSviewer, WoSCC, bibliometric analysis

## Abstract

**Background and aims:**

The tumor microenvironment (TME) has pivotal parts within multiple tumor models of onset/progression, such as triple-negative breast cancer (TNBC). This bibliometric analysis was developed to explore trends and research niches revolving around TME in TNBC.

**Methods:**

Web of Science Core Collection was queried for identifying studies linked with TME in TNBC, after which the VOSviewer, CiteSpace, and R software programs were used to conduct bibliometric analyses and to generate corresponding visualizations.

**Results:**

In total, this study included 1,604 studies published from 2005-2023. The USA and China exhibited the highest numbers of citations, and the research institutions with the greatest output in this field included Harvard University, the University of Texas System, and Fudan University. Ying Wang from Sun Yat-Sen University was the most published and most cited author in this space. The highest number of articles were published in *Cancer*, while the greatest co-citation number was evident in *Breast Cancer Research*. Important keywords related to this research topic included metastasis, tumor-infiltrating lymphocytes, immunotherapy, chemotherapy, and nanoparticles. In particular, pembrolizumab, immunotherapy, nanoparticles, combination treatment, and biomarkers were topics of marked interest in recent reports.

**Conclusion:**

The TME in TNBC is an area of rapidly growing and evolving research interest, with extensive global collaboration helping to drive this field forward. Antitumor therapies targeting the TME in TNBC patients represent an emerging topic of future research, providing opportunities for translational findings. The results of this analysis may provide additional guidance for work focused on the TME in TNBC.

## 1 Introduction

The tumor microenvironment (TME) surrounds tumor cells and consists of fibroblasts, immune cells, blood vessels, extracellular matrix (ECM) components, and a range of signaling molecule types (1, 2). Many studies have documented close relationships between tumors and the TME that dynamically shape tumor growth and development through the control of the secretion of bioactive molecules that can facilitate angiogenesis and immune evasion. Different immune cell types within the TME can also shape the tumor progression, especially in patients with confirmed triple-negative breast cancer (TNBC) (3–5).

TNBC cases account for 10-20% of all breast cancer diagnoses, and these tumors exhibit pronounced heterogeneity (6). The immune microenvironment associated with these tumors includes the expression of high levels of vascular endothelial growth factor and other molecules conducive to tumor growth and invasivity, together with abundant tumor-associated macrophages (TAMs) and tumor-infiltrating lymphocytes (TILs) that play dual roles in driving and restraining TNBC development and progression (7). Efforts to understand the TME associated with TNBC tumors have the potential to aid in the diagnosis, prognostic evaluation, and therapeutic management of this form of cancer. Tumor-associated cells also undergo dynamic changes over the course of tumor progression and metastasis such that the intratumoral and peritumoral landscapes are dynamic and unique for each patient. Several different pathways have been identified whereby the TME can promote or suppress TNBC progression through heterogeneity and plasticity (8–10). There is thus a clear need for qualitative analyses of current research focused on the TME within TNBC to highlight active and future hotspots for scientific investigation.

Bibliometrics, an interdisciplinary science, equips researchers with mathematical and statistical tools for a comprehensive and objective evaluation of a specified research field (11, 12). This analytical approach facilitates a deeper understanding of the evolution and development of specific research trajectories. As it enables a comparative study of contributions from diverse countries, organizations, experts, and publications, it also describes and forecasts the future trajectory of a research topic (13). Bibliometric analysis has been used in various fields of medicine, such as cardiovascular diseases (14), endocrine diseases (14), gastrointestinal tumors (15), and the immune microenvironment (16), and is becoming increasingly important in assessing hot frontiers and formulating guidelines between TME and TNBC.

Here, this study explores the hotspots and frontier trends of TME in TNBC research from 2005 to 2023, and forms corresponding knowledge maps with CiteSpace and VOSviewer. This study provides the latest progress, evolution paths, frontier research hotspots, and future research trends for TME-related research in the basic research and clinical prevention and treatment of TNBC.

## 2 Methods

### 2.1 Search strategies

**Figure 1:**
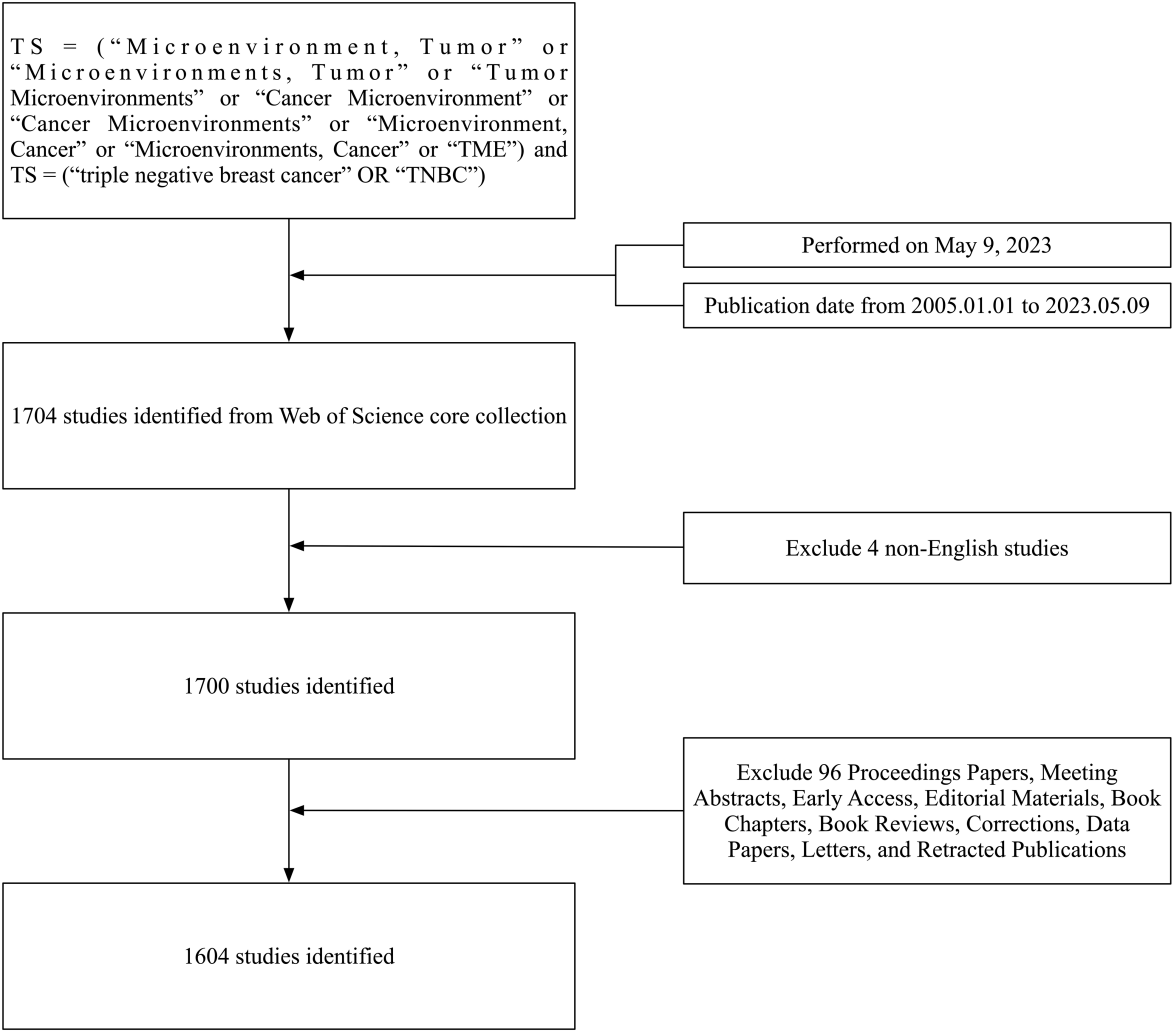
Study search strategy. WoS was searched through terms such as: TS = (“Microenvironment, Tumor” or “Microenvironments, Tumor” or “Tumor Microenvironments” or “Cancer Microenvironment” or “Cancer Microenvironments” or “Microenvironment, Cancer” or “Microenvironments, Cancer” or “TME”) and TS = (“triple negative breast cancer” OR “TNBC”). Only studies published between January 1, 2005 and May 9, 2023 were eligible for inclusion. This approach initially led to the identification of 1,704 studies, four of which were not published in English and were excluded. Subsequently, 1,604 of the remaining studies were deemed eligible for inclusion, while 96 failed to meet the designated inclusion criteria. TS, topic search.

Science Citation Index-Expanded database from Web of Science Core Collection (WoSCC) sourced all data used to conduct this study, which were downloaded on 9 May 2023. The search strategy for this analysis was as follows: Topic Search (TS)= (“Microenvironment, Tumor” or “Microenvironments, Tumor” or “Tumor Microenvironments” or “Cancer Microenvironment” or “Cancer Microenvironments” or “Microenvironment, Cancer” or “Microenvironments, Cancer” or “TME”) and TS = (“triple negative breast cancer” or “TNBC”). Only review articles and studies published in English from 2005 – 2023 were eligible for inclusion, ultimately leading to the identification of 1604 relevant studies (**Figure 1**). Key data for each study (title, publication year, authors, nationality, institutional affiliation, journal, keywords, together with abstract) were downloaded within TXT format through WoSCC and imported within Microsoft™ Excel^®^ for further analysis. All analyses were completed in one day to preclude any effects of database updates on study results.

### 2.2 Data collection

H-index values for individual researchers were obtained through Web of Science, as were journal impact factor (IF)/journal citation report (JCR) values. Productivity was measured based on citation numbers. Any overlapping items were merged, and misspellings were corrected. After data had been cleaned, they were collected for subsequent evaluations.

### 2.3 Bibliometric assessments

Bibliometric indicators provide a means of quantitatively evaluating and summarizing trends within literature for a given field. We used R software to conduct Lotka’s Law analysis (17). The VOSviewer bibliometric tool allows for scientometric network development and the visualization of knowledge (18, 19). VOSviewer-derived network graphs include nodes that are proportional to numbers of publications, with nodes being grouped into clusters based on their relationships with one another. Connecting line girth across nodes indicates associative relationship intensity across both nodes. Centrality values could also quantify value for a given node within an individual network, with critical nodes being those having centrality value > 0.1. The CiteSpace tool also enables the detection of citation bursts as a means of identifying key research hotspots (18). All generated datasets were imported within Microsoft™ Office 365^®^ (WA, USA), VOSviewer (Leiden University, Leiden, The Netherlands), together with CiteSpace V (Drexel University, PA, USA) and were used to conduct the subsequent bibliometric analyses.

## 3 Results

### 3.1 Publication output and temporal trends

**Figure 2:**
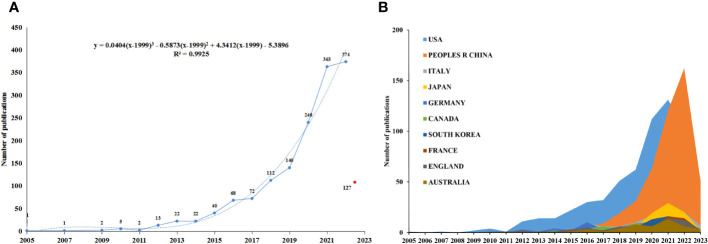
Trends in publication output related to the TME in TNBC by country/region. **(A)** Annual global publication output over time. **(B)** Trends in publication output over time in the 10 most productive countries.

The established search strategy ultimately led to the identification of 1,604 studies (1,387 articles, 217 reviews) eligible for inclusion within present bibliometric analysis. Publication output in this field rose steadily before 2015, after which it rose much more rapidly, such that 374 articles were published in 2022 alone, a nine-fold increase from the 40 articles published in 2015 (**Figure 2A**). A total of 127 articles have been published as of the time this analysis was conducted in 2023. In light of the publication trends, the total number of articles related to the TME in TNBC expected to be published by the end of 2023 is 319.

### 3.2 National contributions and international collaboration

The studies included in this analysis were linked to 2,237 institutions together with 74 nations/areas (see **Supplementary Tables 1**, **2**). The nation with the peak number of publications/citations in this research space was the USA, with 623 (38.840%) articles and 18,951 citations. The USA was followed by China (465 articles; 7,498 citations), Italy (106 articles; 2,886 citations), Japan (92 articles; 2,182 citations), and Germany (70 articles; 2,119 citations). While the USA has remained a dominant contributor to this field, the first published article identified in this analysis was actually from Canada. Although Chinese publications in this space only began in 2014, China had overtaken the USA in annual publication output as of 2022 and rose to the second leading source of publications, underscoring the importance of not underestimating its momentum. The efforts by 10 peak prolific nations/areas are summarized in **Table 1** and **Figure 2B**.

**Table 1:** The top 10 countries/regions and institutions ranked by publication number.

Rank	Country/region	Documents	Citations	Rank	Institution	Documents	Citations
1	USA	623	18,951	1	Harvard University	56	2,393
2	Peoples R China	465	7,498	2	University of Texas System	48	1,443
3	Italy	106	3,984	3	Fudan University	46	836
4	Japan	92	2,886	4	Johns Hopkins University	43	2,096
5	Germany	70	2,119	5	Chinese Academy of Sciences	42	1,449
6	Canada	66	1,569	6	Unicancer	41	1,784
7	South Korea	60	1,443	7	National Institutes of Health USA	38	906
8	France	58	2,056	8	University of Califonia System	38	1,138
9	England	56	1,553	9	Harvard Medical School	36	1,502
10	Australia	46	1,809	10	Udice French Research Universities	36	1,494

**Figure 3:**
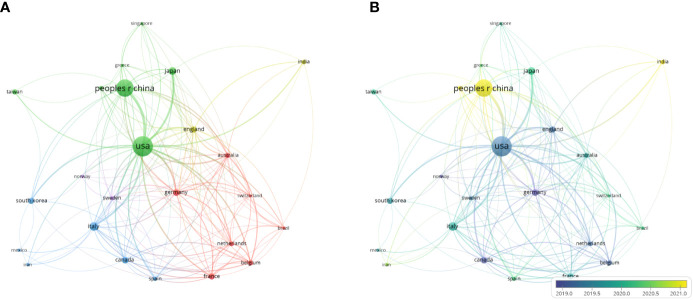
Cluster analyses for nations/areas associated with 15 or more publications. **(A)** A visualization map wherein 24 countries/regions were assigned to 5 clusters based on collaborative interactions. In this diagram, node size was proportional to publication numbers, while colors denote the established clusters, and the thickness of edges is proportional to collaborative intensity between linked nations. **(B)** A visualization map whereby node colors are indicative for mean publication year concerning the specific country/region, from earlier (blue) to more recent (yellow).

A subsequent cluster analysis collected 24 countries/regions with over 15 representations in this dataset within 5 clusters depending upon co-authored article numbers (**Figure 3**, **Supplementary Table 3**). The initial cluster comprised Australia, Belgium, Brazil, France, Germany, Netherlands, together with Switzerland. The second cluster comprised Egypt, Greece, Japan, China, Singapore, Taiwan, and the USA. The third included Canada, Iran, Italy, Mexico, South Korea, together with Spain. The fourth cluster included England and India. The fifth cluster comprised Norway and Sweden. Nations within the first cluster were associated with the greatest links, suggesting China, USA, South Korea, England, and Japan obtain the highest collaborative levels when performing research focused on the TME in TNBC. Substantial bidirectional collaborations between the USA and China were noted in this analysis, with both nations having produced large volumes of mechanistic work related to immunology, cancer development, autophagy and apoptosis, molecular cell biology, and stem cell research, with the latter of these being more clinically oriented. Chinese researchers also tended to focus on subjects including nano-science/-technology, multidisciplinary materials science, surgery, and biomedical engineering. Additional efforts to promote robust international collaborative interactions may drive further advancement of such research.

### 3.3 Institutional research output

**Figure 4:**
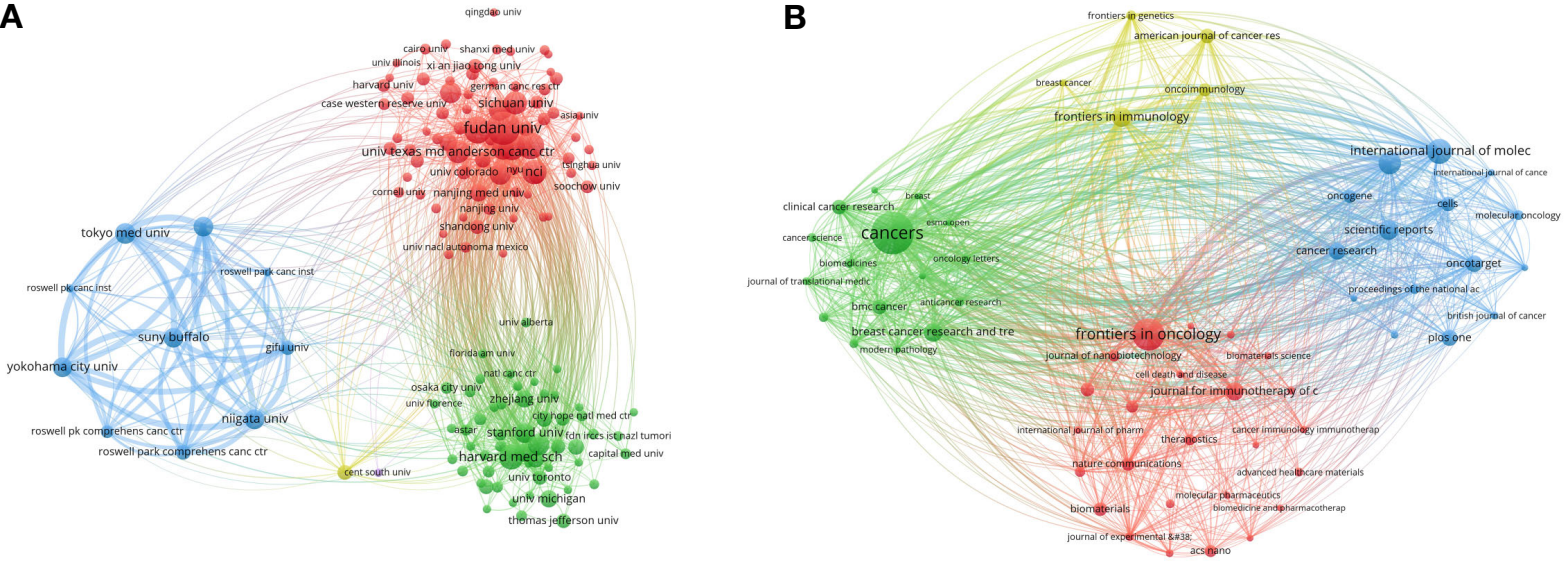
**(A)**The network map of institutions. **(B)**The network map of journals.

The 10 nations that have produced the greatest amount of research in this field included Harvard University (56; 3.491%), the University of Texas System (48; 2.993%), Fudan University (46; 2.868%), Johns Hopkins University (43; 2.681%), and the Chinese Academy of Sciences (42; 2.618%) (**Table 1**). An institutional co-authorship study was conducted with VOSviewer with the goal of more fully exploring collaborations among these institutions (**Figure 4A**; **Supplementary Table 4**). Ultimately, 145 institutions were associated with 6 or more publications and used to establish a co-authorship network composed of 5 clusters, the representative institutions of which included the University at Buffalo–SUNY, Fudan University, Harvard Medical School, Chinese Academy of Medical Sciences & Peking Union Medical College, and Central South University. Of these, the closest collaborative relationships were detected between Tokyo Medical University Hospital, Niigata University, and Yokohama City University.

### 3.4 Leading journals in this field

**Figure 5:**
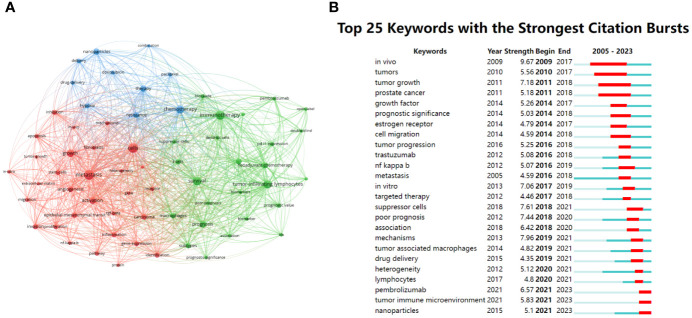
Co-occurrence network visualization map representing the peak 65 keywords. **(A)** Visualization map corresponding to articles related to peak 65 author keywords were collected within three clusters represented by nodes having matching colors. Each keyword reflected a node, with dimension proportional to article quantity. Collaborations are represented by lines between nodes, the thickness of which is proportional to the intensity of relevant association. **(B)** Peak 25 keywords having highest citation bursts pertaining to research focused on the TME in TNBC.

These 1,604 studies focused on the TME in TNBC were published in 454 different scientific journals (**Figure 4B**; **Supplementary Table 5**), including 10-peak representing journals, as listed in **Table 2**. The cited journals network indicated the association between two journals. Journals are divided into four clusters, and the size of nodes represented the number of co-citations (**Figure 5B**). There was a similar theme between journals of the same color, especially for the red cluster. The highest number of articles was published in *Cancers* (97 articles; 6.047%), which had a 2021 IF of 6.575, followed by *Frontiers in Oncology* (64 articles; 3.99%), and *International Journal of Molecular Sciences* (44 articles; 2.743%). Of articles in journals with an IF greater than 10, the most highly ranked included ‘Immune induction strategies in metastatic triple-negative breast cancer to enhance the sensitivity to PD-1 blockade: the TONIC trial’ by Voorwerk and colleagues, and ‘A single-cell map of intratumoral changes during anti-PD1 treatment of patients with breast cancer’ by Bassez and colleagues in *Nature Medicine* (IF = 87.241), followed by ‘Delineating copy number and clonal substructure in human tumors from single-cell transcriptomes’ published by Gao et al. (IF = 68.164). The following three most highly influential articles included ‘Selection of Bone Metastasis Seeds by Mesenchymal Signals within Primary Tumor Stroma’ by Zhang et al., ‘*In vivo* CRISPR screens identify the E3 ligase Cop1 as a modulator of macrophage infiltration and cancer immunotherapy target’ by Wang and colleagues, and ‘A Structured Tumor-Immune Microenvironment in Triple Negative Breast Cancer Revealed by Multiplexed Ion Beam Imaging’ by Keren and colleagues (IF = 66.85; **Table 3**).

**Table 2:** The top 10 journals and authors ranked by publication number.

Rank	Journals	Country	IF 2021	Documents	Citations	Rank	Authors	Documents	Citations
1	Cancers	CH	6.575	97	946	1	Wang Y	24	246
2	Frontiers in Oncology	CH	5.738	64	619	2	Takabe K	21	431
3	International Journal of Molecular Sciences	USA	6.208	44	342	3	Zhang Y	20	373
4	Breast Cancer Research	UK	8.408	37	1132	4	Oshi M	19	415
5	Frontiers in Immunology	CH	8.786	31	361	5	Wang J	19	392
6	Scientific Reports	UK	4.996	29	572	6	Zhang J	19	310
7	Breast Cancer Research and Treatment	USA	4.624	28	791	7	Yan L	17	389
8	Journal of Immunotherapy of Cancer	USA	12.469	27	736	8	Endo I	14	297
9	Cancer Research	USA	13.312	24	942	9	Li J	14	238
10	Plos one	USA	3.752	23	847	10	Tokumaru Y	14	378

**Table 3:** The top 10 authors with the highest impact publications.

Rank	Publication Year	Author	Title	Publication Title	DOI	IF	JCR
1	2019	Leonie Voorwerk; Slagter, Maarten; Horlings, Hugo M.; Sikorska, Karolina; van de Vijver, Koen K.; de Maaker, Michiel; Nederlof, Iris; Kluin, Roelof J. C.; Warren, Sarah; Ong, Sufey; Wiersma, Terry G.; Russell, Nicola S.; Lalezari, Ferry; Schouten, Philip C.; Bakker, Noor A. M.; Ketelaars, Steven L. C.; Peters, Dennis; Lange, Charlotte A. H.; van Werkhoven, Erik; van Tinteren, Harm; Mandjes, Ingrid A. M.; Kemper, Inge; Onderwater, Suzanne; Chalabi, Myriam; Wilgenhof, Sofie; Haanen, John B. A. G.; Salgado, Roberto; de Visser, Karin E.; Sonke, Gabe S.; Wessels, Lodewyk F. A.; Linn, Sabine C.; Schumacher, Ton N.; Blank, Christian U.; Kok, Marleen	Immune induction strategies in metastatic triple-negative breast cancer to enhance the sensitivity to PD-1 blockade: the TONIC trial	Nature Medicine	10.1038/s41591-019-0432-4	87.241	1
2	2021	Bassez, Ayse; Vos, Hanne; Van Dyck, Laurien; Floris, Giuseppe; Arijs, Ingrid; Desmedt, Christine; Boeckx, Bram; Vanden Bempt, Marlies; Nevelsteen, Ines; Lambein, Kathleen; Punie, Kevin; Neven, Patrick; Garg, Abhishek D.; Wildiers, Hans; Qian, Junbin; Smeets, Ann; Lambrechts, Diether	A single-cell map of intratumoral changes during anti-PD1 treatment of patients with breast cancer	Nature Medicine	10.1038/s41591-021-01323-8	87.241	1
3	2021	Gao, Ruli; Bai, Shanshan; Henderson, Ying C.; Lin, Yiyun; Schalck, Aislyn; Yan, Yun; Kumar, Tapsi; Hu, Min; Sei, Emi; Davis, Alexander; Wang, Fang; Shaitelman, Simona F.; Wang, Jennifer Rui; Chen, Ken; Moulder, Stacy; Lai, Stephen Y.; Navin, Nicholas E.	Delineating copy number and clonal substructure in human tumors from single-cell transcriptomes	Nature Biotechnology	10.1038/s41587-020-00795-2	68.164	1
4	2013	Zhang, Xiang H.-F.; Jin, Xin; Malladi, Srinivas; Zou, Yilong; Wen, Yong H.; Brogi, Edi; Smid, Marcel; Foekens, John A.; Massague, Joan	Selection of Bone Metastasis Seeds by Mesenchymal Signals within Primary Tumor Stroma	Cell	10.1016/j.cell.2013.07.036	66.85	1
5	2021	Wang, Xiaoqing; Tokheim, Collin; Gu, Shengqing Stan; Wang, Binbin; Tang, Qin; Li, Yihao; Traugh, Nicole; Zeng, Zexian; Zhang, Yi; Li, Ziyi; Zhang, Boning; Fu, Jingxin; Xiao, Tengfei; Li, Wei; Meyer, Clifford A.; Chu, Jun; Jiang, Peng; Cejas, Paloma; Lim, Klothilda; Long, Henry; Brown, Myles; Liu, X. Shirley	*In vivo* CRISPR screens identify the E3 ligase Cop1 as a modulator of macrophage infiltration and cancer immunotherapy target	Cell	10.1016/j.cell.2021.09.006	66.85	1
6	2018	Keren, Leeat; Bosse, Marc; Marquez, Diana; Angoshtari, Roshan; Jain, Samir; Varma, Sushama; Yang, Soo-Ryum; Kurian, Allison; Van Valen, David; West, Robert; Bendall, Sean C.; Angelo, Michael	A Structured Tumor-Immune Microenvironment in Triple Negative Breast Cancer Revealed by Multiplexed Ion Beam Imaging	Cell	10.1016/j.cell.2018.08.039	66.85	1
7	2022	Bianchini, Giampaolo; De Angelis, Carmine; Licata, Luca; Gianni, Luca	Treatment landscape of triple-negative breast cancer - expanded options, evolving needs	Nature Reviews Clinical Oncology	10.1038/s41571-021-00565-2	65.011	1
8	2020	Kalinsky, K.; Diamond, J. R.; Vahdat, L. T.; Tolaney, S. M.; Juric, D.; O’Shaughnessy, J.; Moroose, R. L.; Mayer, I. A.; Abramson, V. G.; Goldenberg, D. M.; Sharkey, R. M.; Maliakal, P.; Hong, Q.; Goswami, T.; Wegener, W. A.; Bardia, A.	Sacituzumab govitecan in previously treated hormone receptor-positive/HER2-negative metastatic breast cancer: fi nal results from a phase I/II, single-arm, basket trial	Annals of Oncology	10.1016/j.annonc.2020.09.004	51.769	1
9	2018	Buisseret, L.; Pommey, S.; Allard, B.; Garaud, S.; Bergeron, M.; Cousineau, I.; Ameye, L.; Bareche, Y.; Paesmans, M.; Crown, J. P. A.; Di Leo, A.; Loi, S.; Piccart-Gebhart, M.; Willard-Gallo, K.; Sotiriou, C.; Stagg, J.	Clinical significance of CD73 in triple-negative breast cancer: multiplex analysis of a phase III clinical trial	Annals of Oncology	10.1093/annonc/mdx730	51.769	1
10	2016	Conde, Joao; Oliva, Nuria; Atilano, Mariana; Song, Hyun Seok; Artzi, Natalie	Self-assembled RNA-triple-helix hydrogel scaffold for microRNA modulation within tumor microenvironment	Nature Materials	10.1038/NMAT4497	47.656	1

### 3.5 Research productivity by individual authors

A total of 10,309 authors were identified as having contributed to this research niche (**Supplementary Table 6**), with 10 peak prolific published authors, as illustrated in **Table 2**. The most productive author was Wang Y (24 articles), followed by Takabe K (21 articles), Zhang Y (20 articles), Oshi M (19 articles), Wang J (19 articles), and Zhang J (19 articles). Consistent with these results, Wang Y exhibited the highest number of citations at 431. Just 38 individuals authored 11 or more studies in this field, with Wang Y having produced 24 articles associated with 246 citations for an H-index of 9, while work by Takabe K produced 431 citations for the highest overall H-index of 11. Notably, Zhang Y has also published 20 studies to date, with a marked increase in publication output from 2021-2022. All such authors demonstrated >8 H-index. The peak-cited article from such an investigation was by Costa A et al. and was published in *Cancer Cell* (754 citations), followed by an article by Keren L and colleagues (434 citations) and a clinical trial by Voorwerk L and colleagues (425 citations; **Table 4**).

**Table 4:** The top 10 authors with the most highly cited articles.

Rank	Authors	Title	Journal	Year	Citations	IF
1	Costa, A; Mechta-Grigoriou, F	Fibroblast Heterogeneity and Immunosuppressive Environment in Human Breast Cancer	Cancer Cell	2018	741	38.585
2	Keren, L; Angelo, M	A Structured Tumor-Immune Microenvironment in Triple Negative Breast Cancer Revealed by Multiplexed Ion Beam Imaging	Cell	2018	434	66.85
3	Voorwerk, L; Kok, M	Immune induction strategies in metastatic triple-negative breast cancer to enhance the sensitivity to PD-1 blockade: the TONIC trial	Nature Medicine	2019	425	87.244
4	Golden, EB; Formenti, SC	Radiation fosters dose-dependent and chemotherapy-induced immunogenic cell death	Oncoimmunology	2014	360	7.723
5	Wang, C; Gu, Z	*In situ* activation of platelets with checkpoint inhibitors for post-surgical cancer immunotherapy	Nature Biomedical Engineering	2017	337	29.234
6	Wang, T; Semenza, GL	Hypoxia-inducible factors and RAB22A mediate formation of microvesicles that stimulate breast cancer invasion and metastasis	Proceedings of the National Academy of Sciences of the United States of America	2014	335	12.779
7	Pantelidou, C; Shapiro, GI	PARP Inhibitor Efficacy Depends on CD8(+) T-cell Recruitment via Intratumoral STING Pathway Activation in BRCA-Deficient Models of Triple-Negative Breast Cancer	Cancer Discovery	2019	335	12.779
8	Zhang, XHF; Massague, J	Selection of Bone Metastasis Seeds by Mesenchymal Signals within Primary Tumor Stroma	Cell	2013	289	66.85
9	Nedeljkovic, M; Damjanovic, A	Mechanisms of Chemotherapy Resistance in Triple-Negative Breast Cancer-How We Can Rise to the Challenge	Cells	2019	288	7.666
10	Semenza, GL	The hypoxic tumor microenvironment: A driving force for breast cancer progression	Biochimica ET Biophysica ACTA-Molecular Cell Research	2016	282	5.011

### 3.6 WoS sections

Section assessments led to the identification of 69 total categories appearing more than 50 times (**Table 5**), the top 10 of which included the following: Oncology, Cell Biology, Biochemistry Molecular Biology, Pharmacology, Pharmacy, Chemistry Multidisciplinary, Immunology, Medicine Research Experimental, Multidisciplinary Sciences, Nanoscience Nanotechnology, and Materials Science Multidisciplinary.

**Table 5:** Categories represented over 50 times.

Rank	Category	Documents
1	Oncology	734
2	Cell Biology	176
3	Biochemistry Molecular Biology	141
4	Pharmacology Pharmacy	141
5	Chemistry Multidisciplinary	121
6	Immunology	109
7	Medicine Research Experimental	105
8	Multidisciplinary Sciences	101
9	Nanoscience Nanotechnology	101
10	Materials Science Multidisciplinary	68
11	Materials Science Biomaterials	62
12	Pathology	57
13	Engineering Biomedical	55
14	Biotechnology Applied Microbiology	50

### 3.7 Keyword analysis

The VOSviewer tool was subsequently utilized to extract title and abstract keywords from all 1,604 included studies, leading to the identification of 65 keywords that appeared at least 30 times (**Table 6**). Based on their co-occurrence in different articles, these keywords were assembled into three clusters (**Figure 5A**). Of these, the initial cluster (red) comprised 30 keywords, the most used keywords were metastasis, cells, growth, activation, and carcinoma. A second cluster (green) included 25 keywords, the most used keywords were immunotherapy, survival, tumor-infiltrating lymphocytes, prognosis, and neoadjuvant chemotherapy. A third cluster (blue) included 10 keywords, the most used keywords were chemotherapy, therapy, nanoparticles, resistance, and hypoxia.

**Table 6:** Keyword clustering analysis results.

Cluster	Keyword	Rank	Occurrence frequency	Average publication year
1	metastasis	1	251	2019.388
1	cells	2	239	2019.7384
1	growth	7	150	2019.3289
1	activation	9	112	2019.7117
1	carcinoma	14	88	2018.7273
1	angiogenesis	15	86	2018.4884
1	epithelial-mesenchymal transition	17	77	2019.3247
1	invasion	19	74	2019.2432
1	identification	21	71	2019.2429
1	gene-expression	23	68	2019.1324
1	stem-cells	24	67	2019.2273
1	inflammation	26	63	2019.5714
1	inhibition	27	63	2019.3226
1	proliferation	28	61	2019.7333
1	migration	31	54	2019.5741
1	receptor	32	53	2019.8868
1	apoptosis	33	52	2020.6538
1	mechanisms	35	50	2019.94
1	pathway	36	49	2019.1224
1	tgf-beta	40	43	2020.0698
1	fibroblasts	41	43	2019.5814
1	heterogeneity	45	39	2020.5789
1	nf-kappa-b	50	36	2019.5278
1	gene	51	36	2019.4571
1	*in-vitro*	52	36	2018.8333
1	protein	59	32	2019.4194
1	tumor-growth	60	32	2018
1	extracellular-matrix	61	31	2019.9355
1	growth-factor	62	31	2018.9032
1	*in-vivo*	63	31	2016.8667
2	immunotherapy	3	226	2020.991
2	survival	4	191	2019.8191
2	tumor-infiltrating lymphocytes	5	182	2020.1517
2	prognosis	8	114	2019.8829
2	neoadjuvant chemotherapy	12	94	2020.1596
2	pd-l1	13	91	2020.6333
2	subtypes	18	77	2020.08
2	macrophages	20	72	2020.1972
2	t-cells	25	65	2019.7778
2	prognostic value	30	55	2019.7455
2	pd-l1 expression	34	51	2020.4902
2	blockade	37	48	2020.5778
2	suppressor-cells	39	45	2019.9778
2	pembrolizumab	42	43	2021.1395
2	biomarker	46	38	2020.8378
2	association	47	38	2020.2632
2	poor-prognosis	48	38	2019.2973
2	dendritic cells	49	37	2020.1081
2	open-label	53	35	2021.3824
2	pd-1	54	35	2020.5429
2	tils	55	34	2020.9062
2	biomarkers	56	34	2020.3333
2	prognostic-significance	57	34	2019.1765
2	double-blind	64	31	2021.9
2	pathological complete response	65	31	2019.7419
3	chemotherapy	6	173	2020.2012
3	therapy	10	111	2020.3241
3	nanoparticles	11	104	2020.86
3	resistance	16	86	2020.0714
3	hypoxia	22	71	2020.5652
3	delivery	29	61	2020.6833
3	doxorubicin	38	48	2020.8085
3	drug-delivery	43	42	2020.1842
3	paclitaxel	44	42	2019.6905
3	combination	58	33	2020.8438

Trends in keyword bursts were represented in a visual map, with citation bursts being represented in red (**Figure 5B**). Keywords exhibiting citation bursts at earlier time points included *in vivo*, tumor growth, prostate cancer, and growth factor. More recently from 2018-2023, key research keywords have included the following: suppressor cells, poor prognosis, mechanisms, drug delivery, heterogeneity, lymphocytes, pembrolizumab, immunotherapy, and nanoparticles.

### 3.8 Co-cited reference assessment

**Figure 6:**
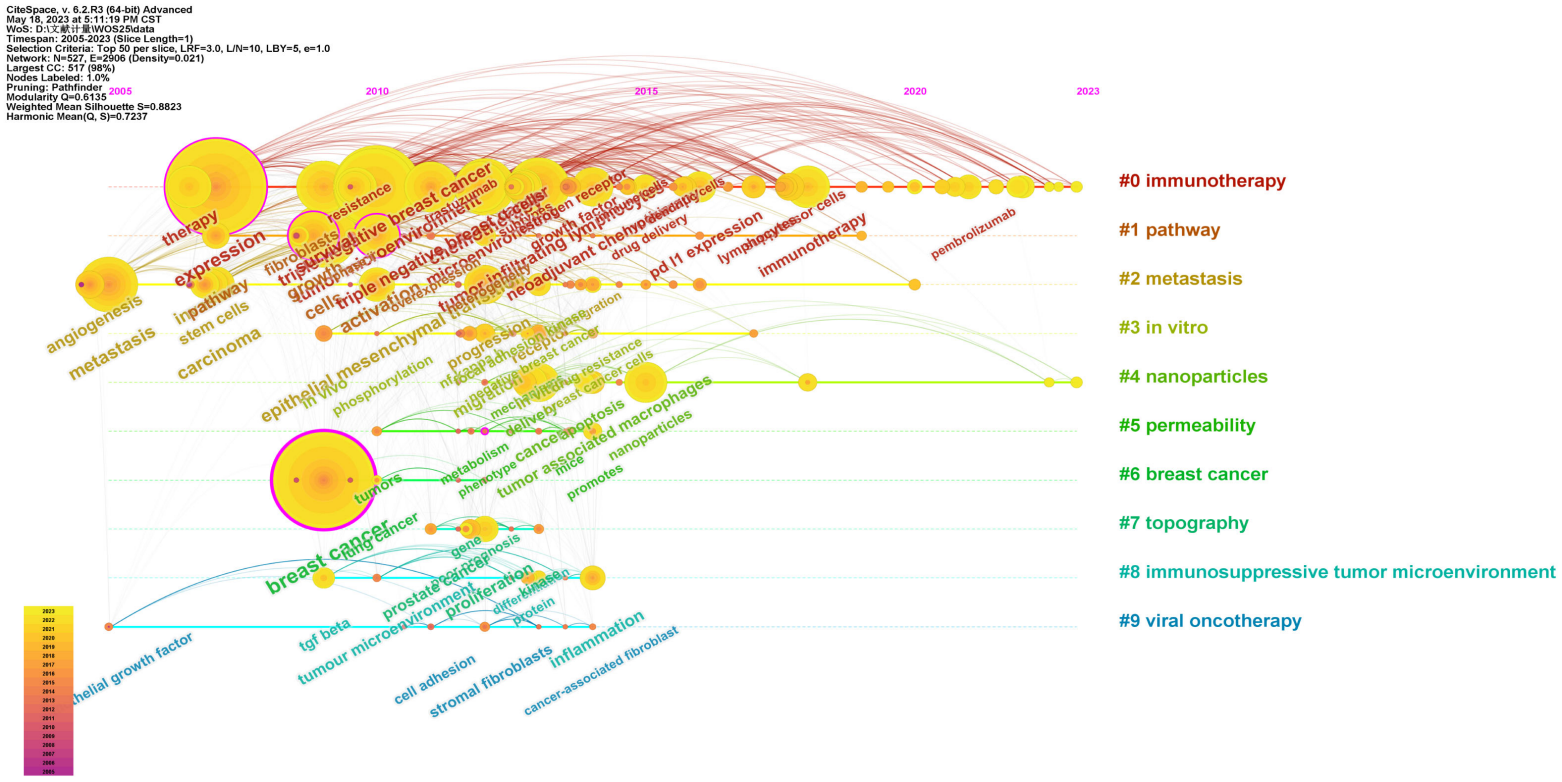
Time-line for co-cited investigations related to the TME and TNBC.

Among 10 peaking co-cited studies identified within the most highly ranked article from Schmid and colleagues (2018), which was classified in cluster 2 and exhibited 200 citations (**Table 7**). Next, a temporal co-citation evaluation was performed (**Figure 6**), revealing predominant relevant studies as published after 2005, with a pronounced increase in publication output from 2015 onward. Initial research niches comprised pathway (cluster #1) and viral oncotherapy (cluster #9) topics, while immunotherapy (cluster #0) was linked to peak publications, emphasizing key importance for TME in studies focused on breast cancer. Immunotherapy (cluster #0), metastasis (cluster #2), and nanoparticles (cluster #4) were identified as the most popular topics in recent years with respect to co-citation output.

**Table 7:** The top 10 co-cited references related to the TME in TNBC.

Rank	Co-citation	Centrality	Author	Year	Journals	Vol	Page	DOI	Cluster
1	200	0.31	Schmid P	2018	New Engl J Med	379	2108	10.1056/nejmoa1809615	2
2	192	0.22	Lehmann BD	2011	J Clin Invest	121	2750	10.1126/science.284.5418.1318	1
3	163	0.19	Salgado R	2015	Ann Oncol	26	259	10.1093/annonc/mdu450	3
4	151	0.11	Dent R	2007	Clin Cancer Res	13	4429	10.1158/1078-0432.ccr-06-3045	1
5	145	0.09	Bianchini G	2016	Nat Rev Clin Oncol	13	674	10.1038/nrclinonc.2016.66	1
6	145	0.11	Foulkes WD	2010	New Engl J Med	363	1938	10.1056/nejmra1001389	1
7	126	0.16	Hanahan D	2011	Cell	144	646	10.1016/j.cell.2011.02.013	1
8	123	0.03	Koboldt DC	2012	Nature	490	61	10.1038/nature11412	1
9	122	0.19	Loi S	2013	J Clin Oncol	31	860	10.1200/jco.2011.41.0902	3
10	119	0.16	Denkert C	2018	lancet oncol	19	40	10.1016/s1470-2045(17)30904-x	2

## 4 Discussion

The TME consists of ECM components and a range of cell types that surround tumor cells, including fibroblasts, endothelial cells, stromal cells, and both innate and adaptive immune cell types. The diverse composition of the TME yields a dynamic and complex network that influences interactions between immune cells and TNBC cells (19). There have been many important research advances focused on the TME in TNBC and other cancer types, and it remains an area of active scientific interest (2, 20). Here, public databases were queried to extract data pertaining to studies focused on the TME in TNBC, after which a bibliometric assessment was performed for pinpointing associated research niches. Such resultant data highlight the key importance of the TME in TNBC.

Analyses of total citation output from specific countries can provide insight into academic status for a specific country within a particular research niche. To date, the USA predominates in research impact concerning TME and TNBC, with 623 publications and 18,951 citations, followed by China (465 publications; 7,498 citations), Italy (106; 3,984), Japan (92; 2,884), and Germany (70; 2,119). Even though the USA remains currently predominant in this field, research output from China continues to grow in both study quality and overall study output such that it will likely surpass the USA within coming years. Indeed, more studies were published in this research space from China than from the USA in 2022 (162 vs.104).

There has been a persistent rising trend in article numbers within such a niche since this topic emerged in 2005. Especially in recent years, the volume of publications has surged, which may be attributed to the technological innovations in this field, such as single-cell sequencing (21). This new appreciation of the biology of TNBC has already led to the development of novel targeted agents, including PARP inhibitors, antibody-drug conjugates and immune-checkpoint inhibitors, which are revolutionizing the therapeutic landscape and providing new opportunities both for patients with early-stage TNBC and for those with advanced-stage disease (22). The countries that first published research in this field were Canada and the USA in 2005 and 2007, respectively. Authors in the USA have published 623 total articles, with 18,951 citations, an H-index of 72, and a citation/article ratio of 30.42, which exceed values of other countries. In contrast, China and Italy, which began publishing research on this topic in 2014, ranked second and third, respectively, in total publication numbers, and exhibited relatively low H-indexes (43 and 29) and citation/article ratios (16.12 and 27.23). While the total research output from China to date in this space is composed of 465 articles with 7,498 citations and both H-index and citation/article ratio values below those for the USA, as of 2022 the annual publication volume from China has surpassed that of the USA, emphasizing the need to pay careful attention to the research momentum of Chinese authors (**Figure 2B**, **Table 1**).

The journals that have published the highest numbers of studies related to the TME in TNBC to date include *Cancers, Frontiers in Oncology, International Journal of Molecular Sciences, Breast Cancer Research*, and *Frontiers in Immunology*. Of these journals, *Cancers* peaked in the number of published articles, followed closely by *Frontiers in Oncology*, together with all top-10 journals were similar in terms of output volume. When assessing IF values, 8 of the top 10 publications with peak impact were published within the previous five years, whereby the topIF value was exhibited by *Nature Medicine* (IF = 87.241, JCR = Q1). *Nature Medicine* could therefore represent a main source of research breakthroughs pertaining to the TME in TNBC within the near future.

The top 10 most productive research institutions in this field were largely consistent with the 10 most productive countries for research output, emphasizing the central role that these institutions serve as facilitators of high-quality academic output. These institutes are expected to produce further breakthroughs relating to the role of the TME in TNBC within coming years. A co-authorship analysis was additionally performed to explore relationships among nations, institutions, and authors, with greater linkage strength suggestive of more robust collaborative interactions. In cluster analyses, the most highly represented institutions included the Chinese Academy of Sciences, Johns Hopkins University, Dana-Farber Cancer Institute, The Netherlands Cancer Institute, University at Buffalo-SUNY, University of Texas MD Anderson Cancer Center, and Weill Cornell Medicine. However, the limited nature of collaborations among these universities highlights the importance of establishing robust relationships that can drive more advanced research in this field in coming years.

The volume of published studies associated with particular topics and categories can provide insight into the major hotspots and areas of research focus in a particular field. The 14 top categories related to the TME in TNBC appeared over 50 times among identified studies, emphasizing growing interest in drugs, immunology, and materials development in this space. The first nanoscience and nanotechnology-related article published in this field, titled “Caging Cancer”, was published in *Nanomedicine - Nanotechnology Biology and Medicine*. This article served as a general overview of cancer-related topics presented for consumption by a general audience, emphasizing the need to treat patients by effectively addressing many different tumor-related factors through appropriately tailored therapeutic strategies in a sequential or simultaneous manner. The chemotherapeutic drugs currently used to treat patients exhibit a high degree of cytotoxicity and only target a single major cancer-related pathway. Nanomedicine-based strategies, in contrast, offer the opportunity to combine multiple therapeutic strategies in a single nanodevice of high complexity. At present, the promise of nanomedicines remains relatively underappreciated, as evidenced by the untapped potential of the gadolinium fullerenol cage molecule Gd@C-82(OH)22. Researchers have demonstrated that this nanomedicine is largely nontoxic and targets multiple tumor-related pathways at the same time, effectively arresting tumor growth even when used to target TNBC cells. These results suggest that developing a more in-depth understanding of the relationships between the physicochemical properties of nanomedicines and biological outcomes in a therapeutic setting may ultimately facilitate the advent of a new approach to reliably treating cancer (20). The first immunity-related article identified in this analysis was published in 2013 in the journal *OncoImmunology* and was entitled “Tumor-infiltrating lymphocytes, breast cancer subtypes and efficacy” (23). This article consisted of an in-depth analysis of over 2000 patient samples derived from a randomized clinical trial, and it ultimately identified a close relationship between high levels of TILs and excellent prognostic outcomes in TNBC patients. In HER2-overexpressing patients, these cells also correlated with better clinical responses to immunogenic chemotherapy, suggesting that immunomodulatory therapeutics may provide a novel avenue for the treatment of patients with aggressive forms of breast cancer (23). In more recent years, a high number of studies focused on this research space such that immune- and material-related therapies are predicted to remain an important focus of active exploration in the future. Multidisciplinary scientific development and ongoing optimization efforts focused on drug efficacy will also inevitably continue over time.

Co-occurrence analyses were also performed with the goal of identifying key topics of research in this field as a means of aiding researchers navigating through various studies. Using keywords derived from study titles and abstracts, a co-occurrence network was established that included three major clusters pertaining to clinically important therapy-/mechanistic-linked studies. Keywords exhibiting the greatest centrality and weight in this network (metastasis, tumor-infiltrating lymphocytes, immunotherapy, chemotherapy, PD-L1, nanoparticles, resistance, and subtypes) are expected to correspond to research niches within this theme, underscoring requirements pertaining to further TNBC-focused research concerning such niches, linked with avenues of investigation. For instance, in recent years, a growing understanding of the molecular mechanisms underlying resistance to taxanes, androgen receptor signaling inhibitors (ARSIs), and poly(ADP-ribose) polymerase inhibitors (PARPi) has emerged. Consequently, strategies to overcome resistance to these therapeutic agents have been developed, significantly enhancing drug efficacy (24). These advancements have notably contributed to the treatment of TNBC.

With the exception of the utilized colors, generated visualization maps appeared identical to the prepared co-occurrence maps, coloring nodes in accordance with mean year of publication for each keyword. Within this analysis, certain keywords exhibited coloration consistent with a greater research focus in recent years (pembrolizumab, immunotherapy, TILs, nanoparticles, combination, and biomarker), indicating that they may be key areas of active research worthy of additional study for researchers seeking to design novel therapeutic approaches targeting the TME in TNBC. Nanotechnology has emerged as a cornerstone in the modulation of the TME, catalyzing the advancement of innovative therapeutic approaches for TNBC. A spectrum of nanoparticles, including silver, gold, and platinum, have been actively incorporated into the treatment modalities for TNBC. State-of-the-art nanoparticle synthesis has been achieved via an array of methodologies, spanning chemical, physical, electrochemical, and biosynthetic techniques. Importantly, the synergy between traditional plant-derived chemotherapeutic agents and engineered nanoparticles has yielded profound anti-cancer properties. These collaborations have been recognized for their environmental sustainability, cost-effectiveness, and enhanced biological efficacy (25). Collectively, these strategies represent promising avenues for the design of novel interventions targeting the TME within the context of TNBC.

The present bibliometric assessment, to the authors’ knowledge, reflects a pioneering focus on research trends pertaining to the TME in TNBC patients. While these results provide a comprehensive high-level overview of research findings in this space with corresponding visualizations of related publications, they are subject to some limitations. For one, the investigation utilized WoS SCIE and excluded any studies not published in English. The significance of more recently published investigations could have been disregarded within a degree as they will inevitably exhibit lower citation frequencies shortly after publication. As bibliometric trends change over time, the conclusions of this study may similarly be altered. Future bibliometric analyses not restricted to English language studies may be warranted in the future.

Together, the present data provide insight into research trends throughout the globe related to research focused on the TME in TNBC. At present, the USA is the predominant contributor to this research niche, and peaking in related studies were published within *Cancers* journal. Clinical studies focused on targeting the TME in this cancer type are potentially a valuable sphere for future research focus, together with present research niches involving studies related to metastasis, tumor-infiltrating lymphocytes, immunotherapy, nanoparticles, chemotherapy, and angiogenesis.

## Data availability statement

The original contributions presented in the study are included in the article/**Supplementary Material**. Further inquiries can be directed to the corresponding authors.

## Author contributions

PL: Writing – original draft. JL: Validation, Writing – original draft. XT: Investigation, Resources, Writing – original draft. ZX: Visualization, Writing – original draft. WD: Data curation, Writing – original draft. CZ: Data curation, Writing – original draft. WW: Writing – review & editing. JZ: Writing – review & editing.

## References

[1] Anderson NM Simon MC. The tumor microenvironment. Curr Biol (2020) 30(16):R921–R5. doi: 10.1016/j.cub.2020.06.081 PMC8194051 32810447

[2] Baghban R Roshangar L Jahanban-Esfahlan R Seidi K Ebrahimi-Kalan A Jaymand M. Tumor microenvironment complexity and therapeutic implications at a glance. Cell Commun Signal (2020) 18(1):59. doi: 10.1186/s12964-020-0530-4 32264958 PMC7140346

[3] Wang Y Chen R Wa Y Ding S Yang Y Liao J. Tumor immune microenvironment and immunotherapy in brain metastasis from non-small cell lung cancer. Front Immunol (2022) 13:829451. doi: 10.3389/fimmu.2022.829451 35251014 PMC8891382

[4] Greten FR Grivennikov SI. Inflammation and cancer: triggers, mechanisms, and consequences. Immunity (2019) 51(1):27–41. doi: 10.1016/j.immuni.2019.06.025 31315034 PMC6831096

[5] Zhao H Wu L Yan G Chen Y Zhou M Wu Y. Inflammation and tumor progression: signaling pathways and targeted intervention. Signal Transduct Target Ther (2021) 6(1):263. doi: 10.1038/s41392-021-00658-5 34248142 PMC8273155

[6] Hubalek M Czech T Muller H. Biological subtypes of triple-negative breast cancer. Breast Care (Basel) (2017) 12(1):8–14. doi: 10.1159/000455820 28611535 PMC5465739

[7] Fan Y He S. The characteristics of tumor microenvironment in triple negative breast cancer. Cancer Manag Res (2022) 14:1–17. doi: 10.2147/CMAR.S316700 35018117 PMC8740624

[8] Mavrommati I Johnson F Echeverria GV Natrajan R. Subclonal heterogeneity and evolution in breast cancer. NPJ Breast Cancer (2021) 7(1):155. doi: 10.1038/s41523-021-00363-0 34934048 PMC8692469

[9] Wu SZ Roden DL Wang C Holliday H Harvey K Cazet AS. Stromal cell diversity associated with immune evasion in human triple-negative breast cancer. EMBO J (2020) 39(19):e104063. doi: 10.15252/embj.2019104063 32790115 PMC7527929

[10] Jaillon S Ponzetta A Di Mitri D Santoni A Bonecchi R Mantovani A. Neutrophil diversity and plasticity in tumour progression and therapy. Nat Rev Cancer (2020) 20(9):485–503. doi: 10.1038/s41568-020-0281-y 32694624

[11] Petcu MA Ionescu-Feleaga L Ionescu B-Ş Moise D-F. A decade for the mathematics: bibliometric analysis of mathematical modeling in economics, ecology, and environment. Mathematics (2023) 11(2):365. doi: 10.3390/math11020365

[12] Liao H Tang M Luo L Li C Chiclana F Zeng X-J. A bibliometric analysis and visualization of medical big data research. Sustainability (2018) 10(2):166. doi: 10.3390/su10010166

[13] Dwivedi YK Kshetri N Hughes L Slade EL Jeyaraj A Kar AK. Opinion paper: “So what if Chatgpt wrote it?” Multidisciplinary perspectives on opportunities, challenges and implications of generative conversational AI for research, practice and policy. Int J Inf Manage (2023) 71:102642. doi: 10.1016/j.ijinfomgt.2023.102642

[14] Wei N Xu Y Wang H Jia Q Shou X Zhang X. Bibliometric and visual analysis of cardiovascular diseases and Covid-19 research. Front Public Health (2022) 10:1022810. doi: 10.3389/fpubh.2022.1022810 36568760 PMC9773213

[15] Yuan F Cai J Liu B Tang X. Bibliometric analysis of 100 top-cited articles in gastric disease. BioMed Res Int (2020) 2020:2672373. doi: 10.1155/2020/2672373 32509853 PMC7245662

[16] Liu YG Jiang ST Zhang L Zheng H Zhang T Zhang JW. Worldwide productivity and research trend of publications concerning tumor immune microenvironment (Time): A bibliometric study. Eur J Med Res (2023) 28(1):229. doi: 10.1186/s40001-023-01195-3 37430294 PMC10332017

[17] Aria M Cuccurullo C. Bibliometrix: an R-tool for comprehensive science mapping analysis. J Informetrics (2017) 11(4):959–75. doi: 10.1016/j.joi.2017.08.007

[18] van Eck NJ Waltman L. Software survey: vosviewer, a computer program for bibliometric mapping. Scientometrics (2010) 84(2):523–38. doi: 10.1007/s11192-009-0146-3 PMC2883932 20585380

[19] Chen C. Science mapping: A systematic review of the literature. J Data Inf Sci (2017) 2(2):1–40. doi: 10.1515/jdis-2017-0006

[20] Balogh LP. Caging cancer. Nanomedicine (2015) 11(4):867–9. doi: 10.1016/j.nano.2015.02.005 25733383

[21] Chen S Zhou Z Li Y Du Y Chen G. Application of single-cell sequencing to the research of tumor microenvironment. Front Immunol (2023) 14:1285540. doi: 10.3389/fimmu.2023.1285540 37965341 PMC10641410

[22] Bianchini G De Angelis C Licata L Gianni L. Treatment landscape of triple-negative breast cancer - expanded options, evolving needs. Nat Rev Clin Oncol (2022) 19(2):91–113. doi: 10.1038/s41571-021-00565-2 34754128

[23] Loi S. Tumor-infiltrating lymphocytes, breast cancer subtypes and therapeutic efficacy. Oncoimmunology (2013) 2(7):e24720. doi: 10.4161/onci.24720 24073365 PMC3782009

[24] Cai M Song XL Li XA Chen M Guo J Yang DH. Current therapy and drug resistance in metastatic castration-resistant prostate cancer. Drug Resist Update (2023) 68:100962. doi: 10.1016/j.drup.2023.100962 37068396

[25] Santhosh SB Shanmugarama S Ramesh N Tharik AMS Basamshetty VV. Recent patents on plant-derived nanoparticles and their potential application towards various cancer therapeutics. Recent Pat Anticancer Drug Discovery (2023) 18(3):292–306. doi: 10.2174/1574892817666220420122426 35450532

